# Role of echocardiography in a patient with suspected acute pulmonary embolism: a case report

**DOI:** 10.1186/s13256-019-1994-y

**Published:** 2019-02-19

**Authors:** Julio Miranda-Bacallado, María Manuela Izquierdo-Gómez, Javier García-Niebla, Juan José Jiménez, José Luis Iribarren, Ignacio Laynez-Cerdeña, Juan Lacalzada-Almeida

**Affiliations:** 1 Cardiac Imaging Laboratory, Department of Cardiology, Hospital Universitario de Canarias, Ofra s/n, La Cuesta, 38320 La Laguna, Tenerife Spain; 2 Servicios Sanitarios del Área de Salud de El Hierro, Valle del Golfo Health Center, El Hierro, Spain; 3 Department of Critical Care, Hospital Universitario de Canarias, Tenerife, Spain

**Keywords:** Pulmonary embolism, Echocardiography, Right ventricular function

## Abstract

**Background:**

Approximately half of pulmonary embolism cases are diagnosed in an emergency context. The classic symptoms of pulmonary embolism are absent in intensive care unit patients who are under sedation and on mechanical ventilation. In this scenario, after the development of sudden, severe hypotension, pulmonary embolism must be considered and included in a differential diagnosis according to the cause of admission. Echocardiography may be of further help in a differential diagnosis of the cause of shock.

**Case presentation:**

We present a case of a 44-year-old Caucasian man who was admitted to the intensive care unit with a diagnosis of community-acquired pneumonia and respiratory failure and who required invasive mechanical ventilation. During admission, the patient developed sudden, severe hypotension that was refractory to treatment. An adequate diagnosis with transthoracic echocardiography was unachievable because of a poor echocardiographic window. However, the combined use of electrocardiography and transesophageal echocardiography established pulmonary embolism as a high-probability diagnosis based on findings of right ventricular pressure overload and right ventricular dysfunction. The unfavorable hemodynamic situation of the patient prevented his transfer to carry out other complementary tests that could confirm the diagnosis of pulmonary embolism. Fibrinolytic and anticoagulant therapies were administered immediately, and a favorable clinical outcome was achieved.

**Conclusion:**

This case highlights the fundamental role that echocardiography played in a patient in the intensive care unit who presented with shock secondary to pulmonary embolism with an unfavorable hemodynamic situation and in whom an unnecessary transfer to perform other complementary diagnostic tests was avoided. The combined use of electrocardiography and echocardiography provided a complete differential diagnosis, identifying the cause of shock and allowing the initiation of specific treatment without further delay. Knowledge of the echocardiographic results that are characteristic of pulmonary embolism can aid in the diagnosis.

**Electronic supplementary material:**

The online version of this article (https://doi.org/10.1186/s13256-019-1994-y) contains supplementary material, which is available to authorized users.

## Background

Approximately half of pulmonary embolism (PE) cases are diagnosed in an emergency context [1–3]. The classic symptoms of PE are absent in intensive care unit (ICU) patients who are under sedation and on mechanical ventilation [4]. In this scenario, after the development of sudden, severe hypotension, PE must be considered and included as a differential diagnosis according to the cause of admission [5]. Our patient was admitted to the ICU with community-acquired pneumonia and was under sedation and mechanical ventilation and suddenly went into shock. In our patient, an adequate diagnosis with transthoracic echocardiography (TTE) was unachievable because of a poor echocardiographic window. Transesophageal echocardiography (TEE) played a pivotal role in our patient’s case, helping with the differential diagnosis of the cause of shock and resulting in a diagnosis of PE as the most prevalent etiology [6]. The robust echocardiographic findings in this case raised the suspicion of PE, avoiding an unnecessary transfer of the patient for other complementary diagnostic tests and justifying the initiation of specific therapy without delay.

In a hemodynamically unstable patient, the mobilization of the patient to perform major complementary tests is difficult to achieve. In this situation, an echocardiographic assessment was available and could be performed, and it played a fundamental role in the diagnosis.

The combined use of TTE and TEE, due to the poor transthoracic window in our patient, ruled out the most common causes of shock: pericardial tamponade, acute valvular dysfunction, severe global or regional left ventricular (LV) dysfunction, aortic dissection, or hypovolemia. The echocardiographic findings of a severely dilated and dysfunctional right ventricle (RV) were confirmed, and focus was placed on a suspected diagnosis of PE. This allowed an early intensive treatment with a favorable outcome for our patient. Knowledge of the characteristic PE echocardiographic findings allowed the diagnosis to be made.

## Case presentation

A 44-year-old Caucasian man, a construction worker in an urban area, married and with two children, with no past medical history, previous treatment, or toxic habits, presented 1 week before entering the hospital with general weakness and respiratory difficulty that gradually increased in intensity, accompanied by cough without expectoration. He had also experienced recent fever (38.9 °C, 102.0 °F) and some episodes of vomiting and diarrhea. He was admitted to the ICU with a diagnosis of community-acquired pneumonia and respiratory failure. At the time of admission to the ICU, the patient was conscious, oriented, and collaborative, without presenting any neurological alteration. The patient was febrile (38 °C, 100.4 °F) and tachycardic (heart rate 110 beats/min), his blood pressure was 120/80 mmHg, and he was tachypneic (28 breaths/min), without intercostal print, with an oxygen saturation of 88% with a Ventimask (Flexicare Medical, Mountain Ash, UK) at 50%. Lung auscultation showed conserved vesicular murmur and basal and midfields bilateral crackles. His heart sounds were regular, rhythmic, and without murmurs. No heart failure data were recorded. We observed a soft and depressible abdomen with peristalsis present, without visceromegalies. The patient’s lower limbs were without edema and had symmetric palpable peripheral pulses. Empiric antibiotic treatment was started with ceftriaxone (2 g/24 h, 7 days), levofloxacin (500 mg/24 h, 7 days), and oseltamivir (150 mg/12 h, 5 days), and 24 h after the admission, the patient was diagnosed with influenza A(H1N1) pneumonia after the virus was isolated in the nasopharyngeal swab samples taken at admission by PCR (DNA isolation). In the patient’s medical history, he did not highlight any history of toxic habits; information on medication taken regularly or any drug allergies was not recorded.

The patient required mechanical ventilation, and his initial evaluation was favorable with stable hemodynamics. On day 12 of the admission, he developed acute severe hypotension (systolic blood pressure < 80 mmHg) with tachycardia (heart rate > 140 beats/min) and a markedly worsening respiratory status. Arterial acid-base balance at that time showed fraction of inspired oxygen 60%, pH 7.39, partial pressure of carbon dioxide 26.7 mmHg, partial pressure of oxygen 55.9 mmHg, bicarbonate 15.9 mmol/L, base excess − 8.1, lactic acid 0.9 mmol/L, and oxygen saturation 91.2%. The patient’s respiratory status failed to respond to high-dose vasopressors and ventilatory support. The laboratory findings at that time showed the following: red blood cells 3.4 × 10^6^/mm^3^, hemoglobin 9.7 g/dl, mean corpuscular volume 96.5 fl (normal reference value 80–100), average corpuscular hemoglobin 28.5, leukocytes 14.8 × 10^3^/mm^3^ (normal reference value 4.5–11.1 × 10^3^), 74.9% neutrophils, 14.8% lymphocytes, international normalized ratio 1.29, basal glucose 155 mg/dl (normal reference value 65–110), blood urea nitrogen 33 mg/dl (normal reference value 5–20), creatinine 1.10 mg/dl, sodium 145 mEq/L, potassium 3.9 mEq/L, troponin I 0.022 ng/dl, (normal reference value < 0.034), D-dimer > 10,000 ng/ml (normal reference value < 500), and C-reactive protein > 90 mg/L (normal reference value 0–12).

**Fig. 1:**
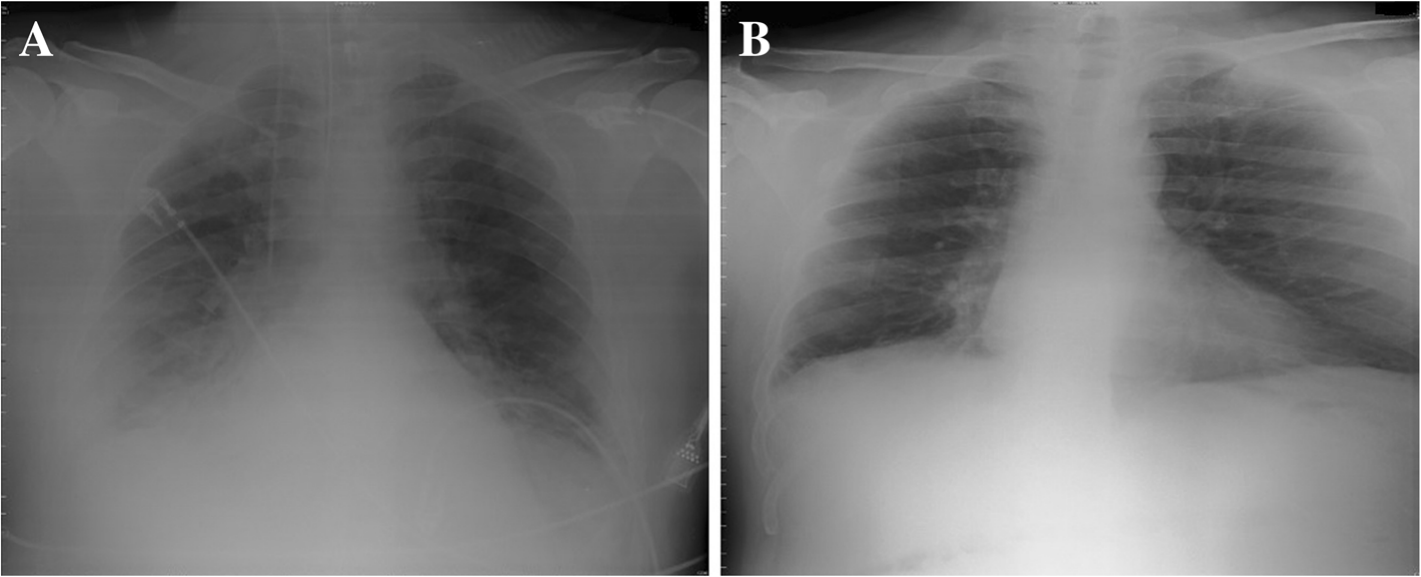
**a** Anteroposterior chest x-ray obtained in the intensive care unit showing basal pulmonary infiltrate. **b** Posteroanterior chest x-ray taken prior to discharge and showing disappearance of the pulmonary infiltrate

The cultures of the bronchial secretion (sputum of the patient) and of urine and blood (direct puncture of a peripheral artery) were negative for both aerobic and anaerobic bacteria, as were urine antigens for *Pneumococcus* and *Legionella*. An anteroposterior chest radiograph showed right basal infiltrate (Fig. 1a). To determine the cause of this acute hemodynamic instability and facilitate patient management, TTE was performed for a differential diagnosis of hypovolemia, acute LV or RV dysfunction, cardiac tamponade, aortic dissection, severe valvular regurgitation, dynamic LV outflow tract obstruction, or PE. Poor-quality images were obtained, necessitating the completion of the study with TEE.

**Fig. 2:**
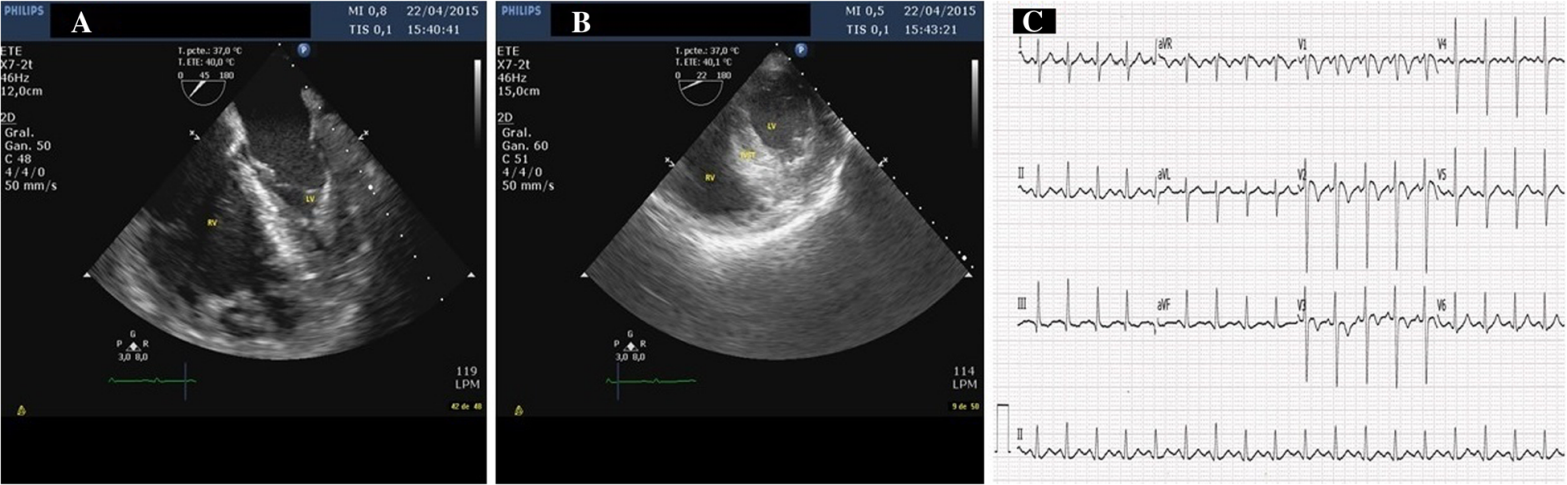
**a** A 45-degree TEE view showing a severely dilated right ventricle with normokinesia of the apical segment and akinesia of the remaining segments of the free wall. **b** Transgastric TEE view showing systolic flattening of the interventricular septum. **c** A 12-lead electrocardiogram shows T-wave inversion in leads V1 to V4 and an S1Q3T3 pattern without abnormalities in the ST segment. *TEE* Transesophageal echocardiography


TEE demonstrated a small and hyperdynamic LV and a severely dilated and dysfunctional RV. In the midesophageal four chambers view with TEE, the RV end-diastolic area to LV end-diastolic area ratio was 1.7 (normal reference value < 0.6), and the RV end-diastolic diameter to LV end-diastolic diameter ratio was 1.4 (normal reference value < 0.9). TEE also showed McConnell’s sign, normokinesia of the RV apical segment, and akinesia of the RV mid-free wall (Fig. 2a, Additional file 1) and a systolic flattening of the interventricular septum (Fig. 2b, Additional file 2), suggesting RV pressure overload. There was no evidence of a thrombus either on the right side of the heart or in the pulmonary arteries. These findings of acute RV failure due to pressure overload raised the possibility of a PE or RV myocardial infarction [1]. A 12-lead electrocardiogram showed T-wave inversion in leads V1 to V4 and an S1Q3T3 pattern without abnormalities in the ST segment (Fig. 2c). The combined use of electrocardiography and TEE in this clinical setting suggested a high probability of PE. The unfavorable hemodynamic situation of the patient prevented transfer to carry out other complementary tests that could confirm the diagnosis of PE. Fibrinolytic and anticoagulant therapies were administered immediately, achieving a favorable clinical outcome.

**Additional file 1:** A 45-degree view of TEE showing severe systolic dysfunction and dilation of the RV, suggestive of pressure overload. *TEE* Transesophageal echocardiography, *RV* Right ventricle, *LV* Left ventricle. (AVI 531 kb)



**Additional file 2:** Transgastric view of TEE showing systolic flattening of the interventricular septum. *TEE* Transesophageal echocardiography, *RV* Right ventricle, *IVST* Interventricular septal thickness, *LV* Left ventricle. (AVI 666 kb)


**Fig. 3:**
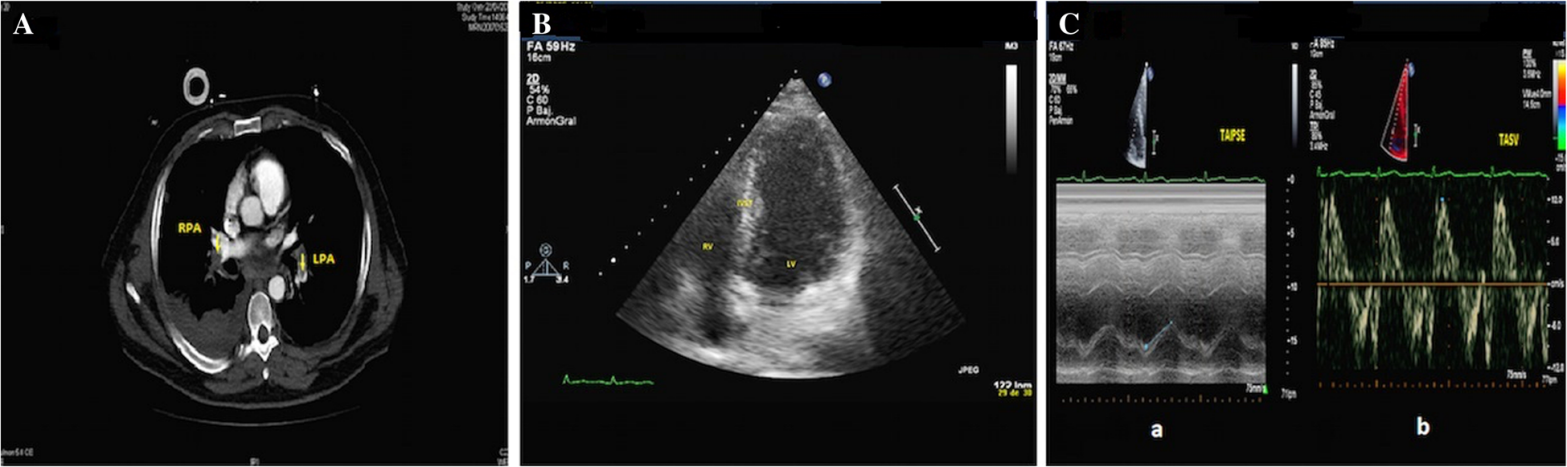
**a** Axial CT slice showing multiple repletion defects in both the pulmonary artery and bilateral lobar arteries, along with peripheral pulmonary consolidations with hypodense zones compatible with areas of hypoperfusion. **b** Four-chamber apical view of TTE showing the right ventricle with normal diameter and contractility after PE treatment. **c** Normal (a) TAPSE and (b) TASV as an expression of functionality of the normal right ventricle after PE treatment. *CT* Computed tomography, *TTE* Transthoracic echocardiography, *PE* Pulmonary thromboembolism, *TAPSE* Tricuspid annular plane systolic excursion, *TASV* Tricuspid annular systolic velocity


Twenty-four hours later, with the patient stable from a hemodynamic and respiratory point of view, computed tomography (CT) pulmonary angiography showed multiple filling defects in both the pulmonary artery and bilateral lobar arteries; this outcome is consistent with PE and peripheral pulmonary consolidations that were more extensive on the right side with hypodense zones compatible with areas of hypoperfusion (Fig. 3a). The diagnosis of PE was confirmed. The patient continued with anticoagulant and antibiotic treatment during admission, progressing favorably from both a hemodynamic and respiratory point of view. Mechanical ventilation was removed on the 27th day. After 11 days of admission, he showed acute renal failure secondary to the nephrotoxic effects of tobramycin, with subsequent normalization of renal function on the 31st day of admission. After completing approximately 2 weeks of rehabilitation, on the 45th day after admission, the patient was discharged without complications. He achieved normalization of the chest x-ray (Fig. 1b) and normalization of RV morphology (Fig. 3b, Additional file 3) and functionality (Fig. 3c). Three years and five months after discharge, the patient remained free of symptoms and was living a normal life.

**Additional file 3:** Four-chamber apical view of TTE showing a morphologically and functionally normal RV after PE treatment. *TTE* Transthoracic echocardiography, *PE* Pulmonary thromboembolism, *RV* Right ventricle, *IVST* Interventricular septal thickness, *LV* Left ventricle. (AVI 833 kb)


## Discussion

This case report illustrates the fundamental role of echocardiography in the ICU for a patient admitted for community-acquired pneumonia with severe shock. The combined use of echocardiography, especially TEE in our patient, with an electrocardiogram, allowed for all possible causes of shock to be quickly ruled out at the patient’s bedside, avoiding unnecessary intrahospital transfers of an unstable patient. In addition, the echocardiographic findings suggestive of PE with repercussion on the RV were recognized, allowing for a suspected diagnosis to be made and for the initiation of fibrinolytic and anticoagulant treatment without further delay, with excellent outcomes in our patient. Although this case is not unique in the literature, it highlights the value of TEE over TTE in patients in the ICU, who, due to the clinical situation (mechanical ventilation, supine position, and so forth), usually have a poor transthoracic window.

Approximately half of PE cases are diagnosed in an emergency setting [1–3]. Dyspnea, chest pain, and syncope are key symptoms that can lead to diagnosis, but these symptoms are absent in ICU patients who are under sedation and on mechanical ventilation [4]. In this scenario, after the development of sudden, severe hypotension, PE must be considered and included in a differential diagnosis according to the cause of admission [5]. TEE played a pivotal role in our patient due to the poor echocardiographic window, which is common in ICU patients. TEE was useful in the differential diagnosis of the cause of shock, ruling out pericardial tamponade, acute valvular dysfunction, severe global or regional LV dysfunction, aortic dissection, or hypovolemia, as recommended by the guidelines of the European Society of Cardiology [6], and resulting in a diagnosis of PE as the most prevalent etiology. Direct visualization of the thrombus is infrequent, and it was not observed in our patient. The echocardiographic diagnosis is based on indirect signs of the physiopathological consequences of increased pressure on the right side of the heart. In addition to TEE findings similar to those of our patient, clinicians may also observe an enlarged pulmonary artery diameter, tricuspid regurgitation that allows for an estimation of the pulmonary artery systolic pressure, an enlarged right atrium, and a dilated inferior vena cava. In the absence of these findings, PE is unlikely [7]. TTE has been established as a valuable tool for evaluating the different causes of hemodynamic instability. Furthermore, TEE has been shown to be of additional value in many instances for critically ill patients because of its ability to provide excellent visualization of cardiac structures. In this context, TTE has a diagnostic success rate of 50% and can occasionally lead to inadequate images that are not able to establish a diagnosis, compared with a 90% success rate for TEE [5]. TEE has 70% sensitivity and 81% specificity for the confirmation of PE [8]. The gold standard for the diagnosis of PE is pulmonary angiography and spiral CT. The PIOPED II trial showed a sensitivity of 83% and a specificity of 96% for multidetector computed tomographic (MDCT) angiography. That trial also highlighted that in patients with a low or intermediate clinical probability of PE as assessed by the Wells rule, a negative CT result had a high negative predictive value for PE (96% and 89%, respectively), whereas this value was only 60% in those with a high pretest probability. Conversely, the positive predictive value of a positive CT result was high (92–96%) in patients with an intermediate or high clinical probability, but much lower (58%) in patients with a low pretest likelihood of PE. Therefore, the PIOPED II trial concluded by warning clinicians to be cautious in cases of discordance between clinical suspicion of PE and MDCT outcome [9]. These techniques require the transport of an unstable patient, which causes a certain risk when performing crucial tests for diagnostic confirmation [5, 10]. In these cases, TEE is a very useful bedside technique for patients in an ICU environment. The recent guidelines of the European Society of Cardiology do not recommend diagnostic echocardiographic studies in patients with suspected (not high-risk) PE. However, signs of RV overload in patients with high-risk PE without another important alternative diagnosis warrant emergency treatment if CT or other confirmatory test results are not immediately available [6]. Thus, although it does not allow an initial PE diagnosis, TEE helps identify when PE is the cause of RV dilation (RV end-diastolic diameter/LV end-diastolic diameter ratio > 0.9) and exclude other causes, such as pericardial effusion or acute myocardial infarction [11]. Echocardiography, on the other hand, has the noninvasive ability to evaluate and monitor the RV and LV function. It also allows the serial determination of different measures of ventricular function, analyzing its response to medical interventions such as fluid and drug therapy. In the specific case of a patient with PE, it is useful for monitoring RV function and pulmonary artery systolic pressure when thrombolytics are administered [12]. In patients with massive PE, serial assessment of RV size and fractional area change, determination of RV systolic pressure, and inferior vena cava assessment can be performed using the American Society of Echocardiography RV guidelines for the normal ranges [13].

## Conclusion

Our patient’s case highlights the fundamental role of echocardiography in critically ill patients for whom the poor-quality imaging of TTE is addressed by TEE. In our patient, TEE allowed us to exclude other alternative diagnoses that were contemplated *a priori* at the bedside of a critically ill patient with severe respiratory and hemodynamic instability. The robust echocardiographic findings in our patient caused us to suspect PE, avoiding an unnecessary transfer of the patient to perform other complementary diagnostic tests such as CT pulmonary angiography and allowing the immediate initiation of specific therapeutics, leading to an excellent clinical outcome. CT pulmonary angiography could be performed later with a more grounded suspicion to justify the initiation of specific therapy. The subsequent TEE examination allowed us to evaluate the effectiveness of medical therapy and possible morphofunctional sequelae on the right side of the heart in the early stage. Therefore, in a patient in the ICU in shock, echocardiography plays a fundamental role in the differential diagnosis of the causes of shock, and knowledge of the echocardiographic findings of PE can prevent the diagnosis from going unnoticed.

## Abbreviations

CT: Computed tomography
ICU: Intensive care unit
LV: Left ventricular
MDCT: Multidetector computed tomography
PE: Pulmonary embolism
RV: Right ventricular
TEE: Transesophageal echocardiography
TTE: Transthoracic echocardiography
